# Providing value-based care as a physiotherapist

**DOI:** 10.1186/s40945-021-00107-0

**Published:** 2021-04-20

**Authors:** Chad E. Cook, Thomas Denninger, Jeremy Lewis, Ina Diener, Charles Thigpen

**Affiliations:** 1 Department of Physical and Occupational Therapy, Duke University Health System, 3475 Erwin Rd, Durham, NC 27705 USA; 2 Department of Orthopaedic Surgery, Duke University Medical Center, 3475 Erwin Rd, Durham, NC 27705 USA; 3 Duke Clinical Research Institute, Duke University, 200 Morris Street, Durham, NC 27701 USA; 4 Duke University, 317 Trent Drive, Durham, NC 27710 USA; 5 ATI Physical Therapy, Greenville, SC USA; 6 School of Health and Social Work, University of Hertfordshire, Hatfield, Hertfordshire AL10 9AB UK; 7 Therapy Department, Central London Community Healthcare National Health Service Trust, London, UK; 8 Department of Physical Therapy & Rehabilitation Science, College of Health Sciences, Qatar University, Doha, Qatar; 9 Stellenbosch University, Stellenbosch, South Africa; 10 University of the Western Cape, Cape Town, South Africa; 11 Physiotherapy clinician, Stellenbosch, South Africa; 12 Center for Effectiveness Research in Orthopaedics, Greenville, SC USA

**Keywords:** Value based care, Physiotherapy, Musculoskeletal disorders, Low Back pain

## Abstract

**Background:**

Despite millions spent in research funding, studies, and guidelines, outcomes involving musculoskeletal care continue to decline. The purpose of this Viewpoint is to describe value-based care and to suggest measures for its adoption by physiotherapists who manage individuals with musculoskeletal related pain disorders.

**Discussion:**

The provision of value-based care is best defined as care that includes: 1) patient centeredness, 2) guideline-oriented, integrated strategies, 3) measurement of patient outcomes and experiences, and 4) cost effectiveness. Physiotherapists are well positioned to be leaders in the application of value-based care by assuring they address each of the four strategies during the daily patient encounter. This Viewpoint discusses strategies for application to clinical practice.

**Conclusion:**

By implementing value-based care principals, physiotherapists could assure that patients with musculoskeletal related pain disorders receive the right care at the right time, by the right provider.

## Introduction

Worldwide, musculoskeletal-related pain disorders (MSK pain disorders) make up three of the top 10 conditions associated with global disability and account for the greatest proportion of persistent pain across all ages and geographies [1]. MSK pain disorders’ incidence levels have increased over the last 30 years and in the United States accounts for direct/indirect medical costs and of USD $874 billion dollars in 2014 [2]. Since 1990, disability associated with LBP, the most common form of MSK pain disorder, has increased by more than 50%, especially in low-income and middle-income countries [1]. Despite “investment” in research funding, numerous clinical practice guidelines, and hundreds of thousands of publications, overall outcomes have not improved over the last decade [3]. This stagnancy in outcomes has coincided with alarming increases in costs.

A different approach to MSK management is required to address these shortcomings, including a re-imagining of best practice implementation that includes perspectives from the patient, clinician, community, and the healthcare institution. The re-imagining should improve the overall “value” of care, by assuring that the right provider delivers the right care at the right time. We feel that physiotherapists are uniquely qualified to lead in the provision of value-based care for these conditions [4]. In this viewpoint, we will describe the constructs associated with value-based care and suggest measures of adopting value-based care principles in day-to-day clinical practice. We aim to demonstrate that the traditional definition of value-based care, which has focused only on costs and outcomes, is too limited. We argue that including principles such as high-level, guideline concordant management and involving the patient in decision-making, are also key elements of value-based care [5].

## Defining value-based care

There are inconsistencies in determining whether value-based care is an *outcome* (i.e., cost effectiveness) or a *description of care services* (i.e., who provided care and whether it matched guidelines). Only recently, has the concept shifted to represent a *description of care services*, a shift that allows suggestions of best practice and opportunities to change. This includes changing from a volume-based care service (being reimbursed by how many people you see) to a value-based care service (being reimbursed based on the quality of your care).

**Fig. 1:**
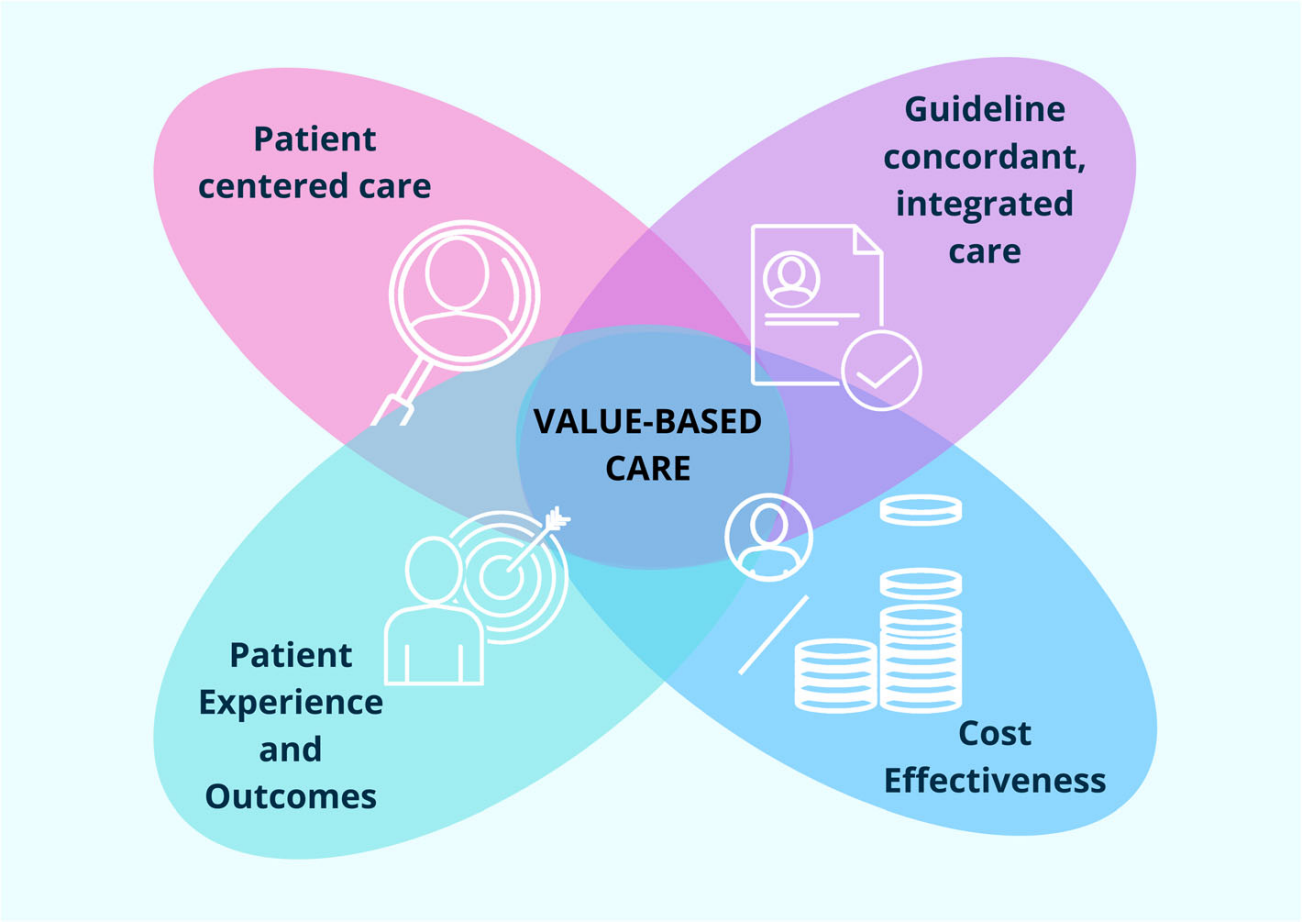
The four components of value-based care

There is poor alignment with the competition seen in fee for service-based health care and elements that are meaningful to patients. For providers, payment in a value-based care model rewards those who deliver care that is efficient and effective, such as those who avoid unnecessary costly interventions or those associated with long-term addiction or substance abuse. Intuitively, value requires the patient as an active contributor to the definition. Similarly, because the relationship between treatment and outcomes is tenuous, a careful evaluation of how the patient was managed (who they saw, when they were seen, and what they were told) requires careful attention. We argue that providing value-based care services requires the clinician to consider: 1) patient centered care, 2) guideline concordant, integrated care, 3) both patient outcomes and experience, and 4) cost effectiveness (Fig. 1).

### Patient centered care

Patient-centered care is the practice of caring for patients (and their families) in ways that are respectful of, and responsive to, individual patient preferences, beliefs, needs and values, and ensuring that patient values guide all clinical decisions [6]. Patient-centered care is framed around the moral obligation to care for patients on their own terms, in their social context, while meeting their needs, (e.g., hours of operation, using telehealth or direct access options, and care that is transferable to a self-management setting) not yours. Patient-centered care goes beyond the clinician-patient encounter and is designed to promote collaborative decision-making.

### Guideline concordant, integrated care

Clinical practice guidelines (CPGs) are a repository of recommended interventions evaluated in past-randomized clinical trials. CPGs reduce clinical care variability and improve the likelihood that the patient receives a treatment that has a measurable clinical effect. Integrated care emphasizes both the “timing” and “the recommended care giver” who provides care [7]. Optimizing the timing of care can lead to improved outcomes and results in better patient experiences and reductions in future costs [8]. Newly integrated care models, such as stepped care or stratified care assist in determining appropriate frequency of care and limit exposure to providers who specialize in invasive, expensive care options that are germane for a small proportion of the patients with MSK pain disorders.

### Patient experience and outcomes

Patient experience encompasses the range of interactions that patients have with the health care system, the care they received, and the challenges they had during scheduling, transportation or payment. Health outcomes may reflect many constructs (e.g., function, pain, and quality of life) and are captured in numerous ways (i.e., self-report, physical performance). Health outcomes are influenced by many factors outside of the treatment provided, including patient experiences; patients who report good experience with their care also have better short- and long-term outcomes [9].

### Cost effectiveness

Costs are an administrative measure, which indirectly assesses the intensity and complexity of care utilization. At face value, lower costs are inherently associated with value-based care; however, this is not as direct as individuals may assume. Further complicating issues is the fact that reimbursement systems differ from country to country, making direct comparisons a challenge. Costs are a historical measure of value-based care, but require consideration within the context of other factors, including the care provided and the patient engagement and experience.

## Practical application for physiotherapists

Adopting value-based care principles in clinical practice is germane to all physiotherapists, regardless of their reimbursement systems. Application of a value-based approach is complicated, one that requires creating a partnership with each patient to identify the most meaningful and effective approach. Below, we discuss adoption of the four key value-based care principles by framing these around three questions: *1) Do I know if my patients are getting better; 2) Does my clinical care reflect best practice guidelines; and 3) what opportunities do I have to move toward value-based care models?*; which are also summarized in Table 1.

**Table 1:** Key Value-Based Principle to Include in Clinical Practice

Concept	Methods to Adopt in Practice	Reasoning
Patient centered care	• Recognize that the needs and preferences of each patient are unique • Use patient decision making support tools	• Patient Expectations, a critical element in patient centered care, are primary drivers to outcomes for many MSK pain conditions. • Patient decision-making support tools allow a reconciliation between the patient and clinician.
Guideline concordant, integrated care	• Be aware of clinical practice guidelines for dedicated areas of care • Refresh your knowledge of integrated care models such as Stepped Care and Stratified Care approaches.	• Clinical practice guidelines focus on interventions that have been shown to have a meaningful clinical effect on the condition they represent. • Stepped care is designed to address appropriate timing, order, and frequency of care (low risk/cost before high risk/cost), which limits care from providers who provide unnecessary early invasive, expensive care options. Use of stepped care for individuals with MSK pain conditions has been shown to reduce overall costs, thus increasing value. • Risk-stratification identifies patients in high distress who needs immediate psychologically informed physiotherapy, and may lessen treatment time and speed up return to work.
Patient Experience and Outcomes	• Routinely capture outcomes such as Patient-Reported Outcomes Measurement Information System (PROMIS) measures or the Single Alpha Numeric Evaluation (SANE) • For patient experience, routinely capture the Consultation and Relational Empathy (CARE) or the Patient Perception of Patient-Centeredness (PPPC) questionnaire	• Patient reported outcomes are designed to reflect the patient’s current health status. By using different constructs (e.g., function, pain intensity, and pain interference), the clinician can better understand the patient’s overall progress in their recovery. • Patient reported experience measures (PREMS) capture patients’ interactions with healthcare systems and the degree to which their needs are being met. PREMS often expose areas of the patient-clinician interaction that are poorly understood but are meaningful.
Cost Effectiveness	• Reduce the use of productivity models that reward productivity and volume • Adopt models that are tied to correct services and outcomes	• Models that reward increased productivity are inclined to include unnecessary treatment or lower value care. • Models that are tied to outcomes or “correct practice patterns” will still be profitable for the clinician, but will also yield positive overall results.

### Do I know if my patients are getting better?

Physiotherapists should routinely capture information that patients’ value, such as functional outcomes and patient experience. For functional outcomes, we recommend a weekly capture of pain intensity, pain interference, and disability/function, and advocate the use of short but valid instruments that are readily available and have strong content validity. Once data are collected, we suggest analyses in a meaningful manner that provides useful information for the clinic and the clinicians but also a discussion of the findings with the patient. Data driven decisions will assist in recognizing gaps in care and may provide opportunities to identify patterns in patients who do not improve. It also promulgates a culture in which the patient contributes to his or her own assessment (via outcomes measures) in a systematic manner.

### Does my clinical care reflect best practice guidelines?

Using CPGs as an overarching guidance of care provided should reduce care variation and costly unnecessary interventions. To determine if clinical care best reflects CPGs, a careful audit of treatment provided is necessary on a routine basis. Patient centered care is a recommendation of most CPGs, thus evaluating whether care preferences of the patient were “matched” with the care provided is an additional recommended step.

### What opportunities do I have to move toward value-based care models?

Our first suggestion involves incorporating patient-centered strategies for care. This involves focusing on patient needs, understanding the resources they have for continued self-management and matching care preferences. In situations involving indecision, patient-decision making support tools such as option grids and decision boxes allow the patient and the clinician to negotiate a treatment approach they each finds valuable. When used correctly, these tools may empower the patient towards taking responsibility and self-management of their condition.

Our second suggestion involves a focus on improving processes of care. If the right providers give the right care at the right time, it should optimize outcomes and reduce future costs that occur when the condition is less malleable to conservative management. Focusing solely on costs has had unintended or unbalanced consequences (cost savings occurred but with worse outcomes) [10]. Hypervigilance in cost reduction often results in equivocal or ‘non-inferior’ outcomes for patients, with less control over care delivery for providers.

Our last suggestion involves the careful reflection of whether a physiotherapist is the best provider for that patient. Although the scope of practice of a physiotherapist has widened markedly, patients with significant trauma, notable psychological disorders, or neuropathic conditions are best managed pharmaceutically, or co-managed with another provider. Placing the patients’ needs first will always drive value-based care.

## Conclusion

Value-based care principles reflect the ability to provide the best care, which results in the best experiences and outcomes, at a reasonable financial investment. Physiotherapists have a unique opportunity to be at the forefront as a value-based provider, especially for MSK related pain disorders. By implementing value-based care, physiotherapists can assure that the right patient receives the right care at the right time, by the right provider.

## Data Availability

(NA, no data)
